# Supplementary material for: Emergence of self-affine surfaces during adhesive wear
Source: Nat Commun. 2019 Mar 8;10:1116. doi: 10.1038/s41467-019-09127-8 (PMC6408517; doi:10.1038/s41467-019-09127-8)
Supplement: Supplementary file 1 — Supplementary Information [file 41467_2019_9127_MOESM1_ESM.pdf]

Supplementary Information
"Emergence of self-affine surfaces during adhesive wear"

E. Milanese[1], T. Brink[1], R. Aghababaei[2], J.-F. Molinari[1,*]

[1]Civil Engineering Institute, Materials Science and Engineering Institute,
École Polytechnique Fédérale de Lausanne (EPFL), CH-1015 Lausanne, Switzerland
[2]Department of Engineering - Mechanical Engineering, Aarhus University, 8000 Aarhus C, Denmark
[*]corresponding author: jean-francois.molinari@epfl.ch

Supplementary figures

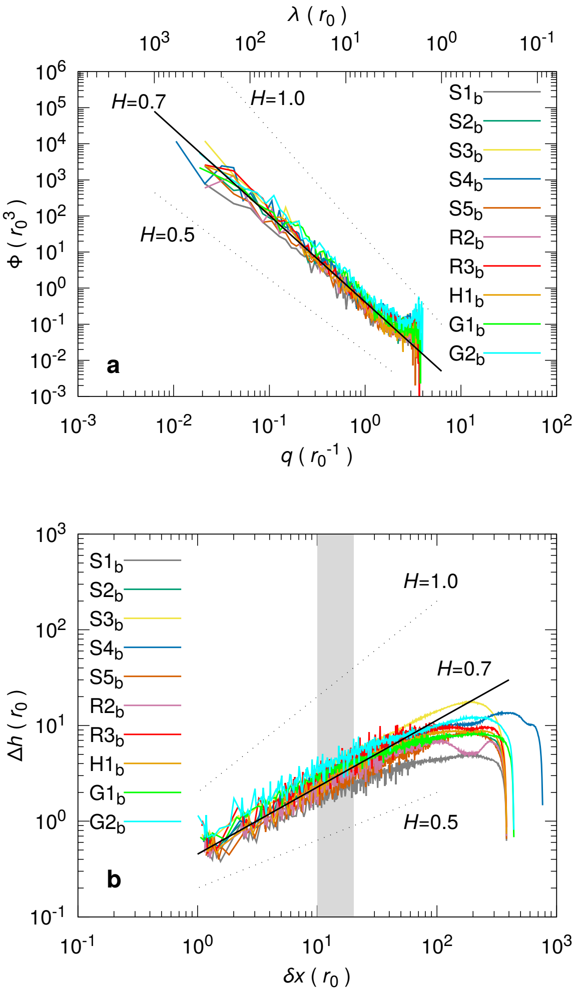

Supplementary Figure 1: Steady-state surface morphology analysis. a) PSD per unit length Φ as a function of the wavevector q and the wavelength λ, the relation between the two being $q = 2\pi/\lambda$. b) Height-height correlation function $\Delta h(\delta x) = \langle [h(x+\delta x) - h(x)]^2 \rangle^{1/2}$. The surfaces are taken from ten different simulations (see Table 1 for details), the subscript indicates the bottom surface for each simulation. Top surfaces for the same simulations are reported in Figure 2. In both a) and b) the solid black straight guide-line corresponds to a Hurst exponent $H = 0.7$. Dotted black straight guide-lines show the hypothetical slope for distributions of $H = 0.5$ and $H = 1.0$. In b) the shaded area displays the interval of distances corresponding to the range of critical length scale values d^* exhibited by the adopted potentials. No pronounced crossover is exhibited in the slope of $\Delta h(\delta x)$ over the range of values for d^*. As a consequence of the assumption of periodic surfaces, the function is roughly symmetric with respect to half the horizontal box size (hence the plateau and the following drop for large values of δx).

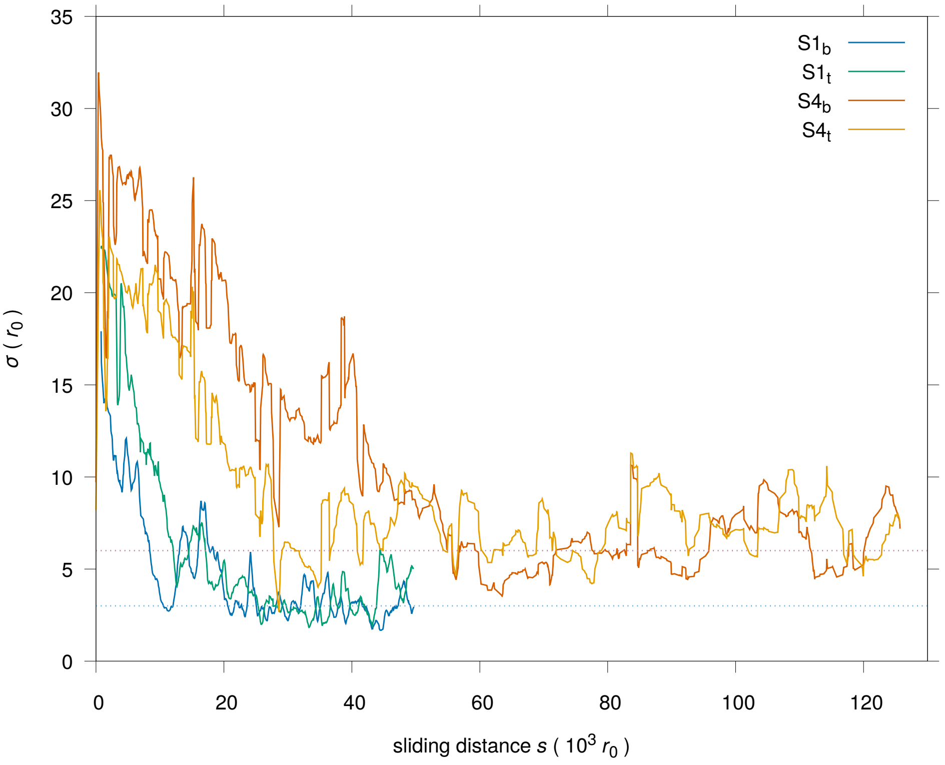

Supplementary Figure 2: Scaling of the root mean square of heights σ with system size. See Methods for the definition of σ. Surfaces are taken from the large simulation S4 and from simulation S1, which is run under the same conditions but has half the horizontal box size than simulation S4 (cf. Table 1). The different steady-state values are consistent with the size dependency of σ. In a first approximation, assuming the same Hurst exponent, the ratio between the values of σ of two surfaces is $\frac{\sigma_{\text{new}}}{\sigma_{\text{ref}}} \approx \left(\frac{\lambda_{\text{new}}}{\lambda_{\text{ref}}}\right)^H$, where λ_{new} is the largest wavelength represented by the surface for which we do not know σ and λ_{ref} is the largest wavelength of the reference surface for which σ is known. It is reasonable to assume that the largest wavelength is proportional to the system size in our case. Assuming $H = 0.7$, the expected ratio for our simulations is approximately 1.5. In the figure the straight horizontal dotted lines corresponds to $\sigma = 3\ r_0$ and $\sigma = 6\ r_0$ respectively, which gives a ratio of 2. While the latter is larger than expected, the order of magnitude is comparable and acceptable, given the noise in the data and the assumption that the Hurst exponent is the same for the two surfaces.

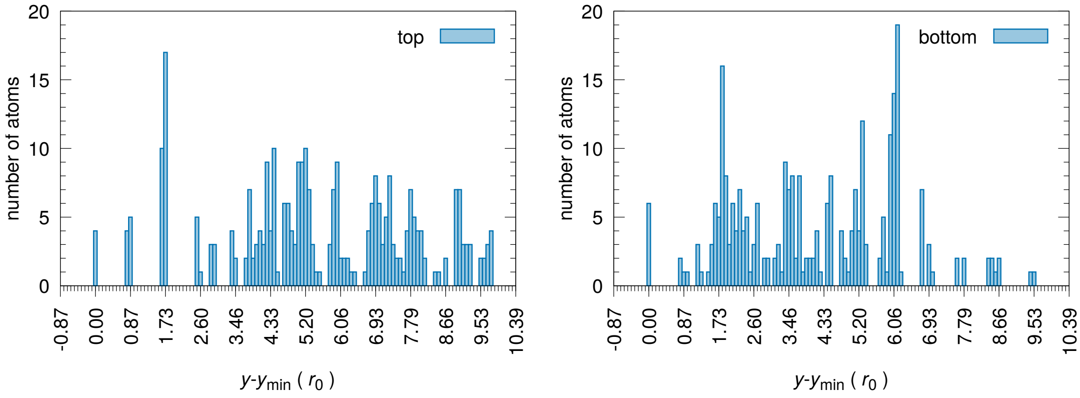

Supplementary Figure 3: Height distribution for top (left) and bottom (right) surfaces of simulation S1 at sliding distance 29 950 r_0, as in Figure 1d, after minimization to avoid effects due to temperature fluctuations. The minimum height $y_{\rm min}$ in the data set is subtracted from each point's y coordinate. Heights are distributed in bins of size $0.1 \cdot \sqrt{3}/2\ r_0$, corresponding to one tenth of the lattice spacing in the y direction. Values in labels along the x axis are in multiples of $\sqrt{3}/2\ r_0$; subtics are $0.1 \cdot \sqrt{3}/2\ r_0$ apart. The spread of the heights shows that the lattice planes are not aligned across the sample and confirms that deformation mechanisms (such as dislocations) play a role in the roughening of the surface.

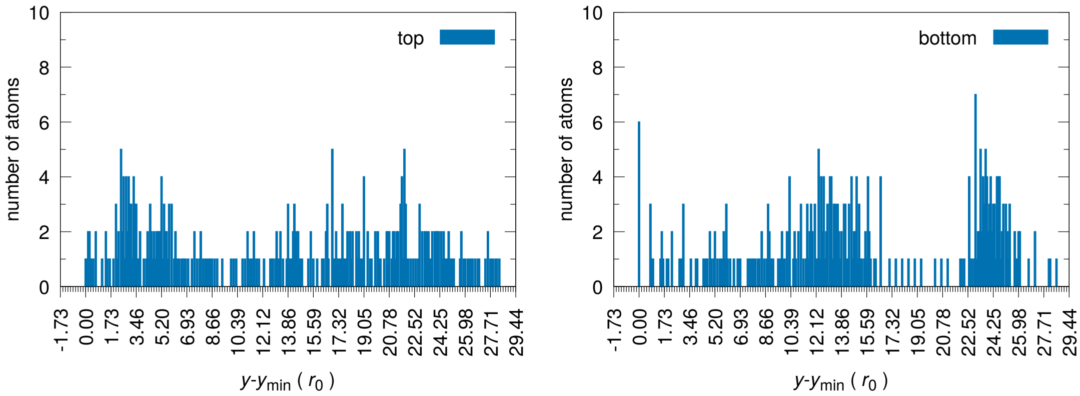

Supplementary Figure 4: Height distribution for top (left) and bottom (right) surfaces of simulation G1 at sliding distance 29 750 r_0, as in Figure 1p, after minimization to avoid effects due to temperature fluctuations. The minimum height $y_{\rm min}$ in the data set is subtracted from each point's y coordinate. Heights are distributed in bins of size $0.1 \cdot \sqrt{3}/2\ r_0$, corresponding to one tenth of the lattice spacing in the y direction. Values in labels along the x axis are in multiples of $2 \cdot \sqrt{3}/2\ r_0$; subtics are $0.2 \cdot \sqrt{3}/2\ r_0$ apart. The spread of the heights shows that the lattice planes are not aligned across the sample. In this case, where grain boundaries are modelled, the effect is even more pronounced (cf. height distribution for single crystal simulation in Supplementary Fig. 3). Grain rotation, in fact, provides an additional mechanism for the surface roughening, besides those already present in the single crystal case (such as dislocations).

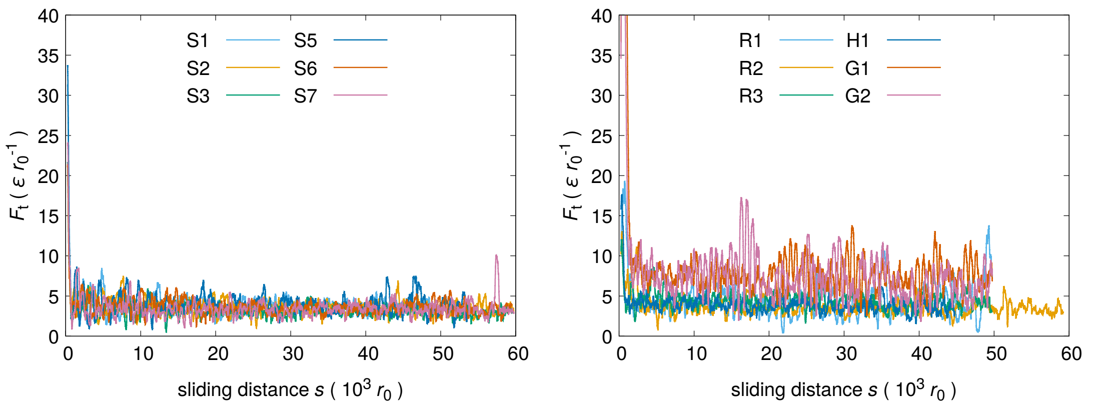

Supplementary Figure 5: Evolution of tangential force F_t with the sliding distance s. Left: the single-asperity simulations, right: all other simulations. After initial peaks due to the debris particle formation [1], F_t oscillates around a steady-state value. Simulation R1 displays large local oscillations due to re-roughening of the surface (cf. Figure 3). Simulations G1 and G2 globally display larger oscillations as a consequence of the presence of grain boundaries (cf. Figure 9).

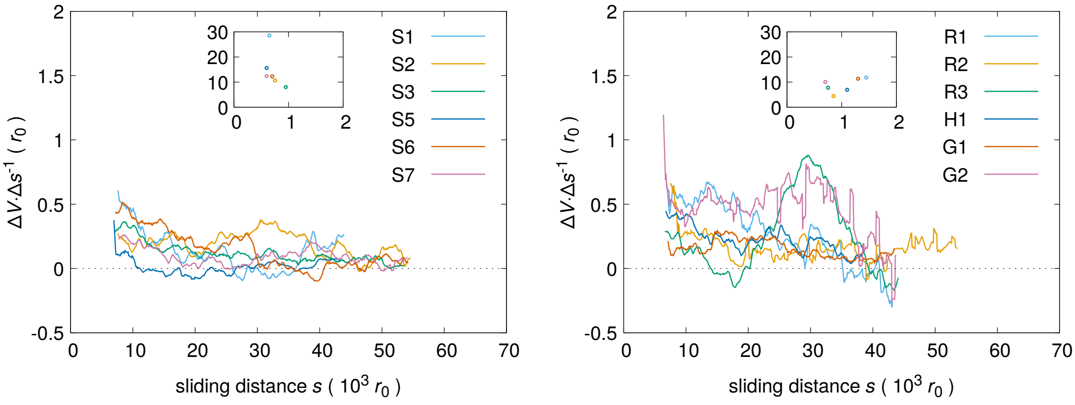

Supplementary Figure 6: Evolution of the wear rate with the sliding distance s. The wear rate is expressed as the ratio between the change of volume ΔV and the change of sliding distance Δs for the single-asperity simulations (left) and all others (right). The insets show the value of $\Delta V/\Delta s$ upon debris particle formation. In all cases the wear rate decreases from a high initial value due to particle formation (inset) to lower values (main plot), indicating two distinct regimes corresponding respectively to two- and three-body sliding configurations. The dotted, black, straight guide-lines show the null wear rate, i.e. the debris particle would neither accumulate nor lose volume.

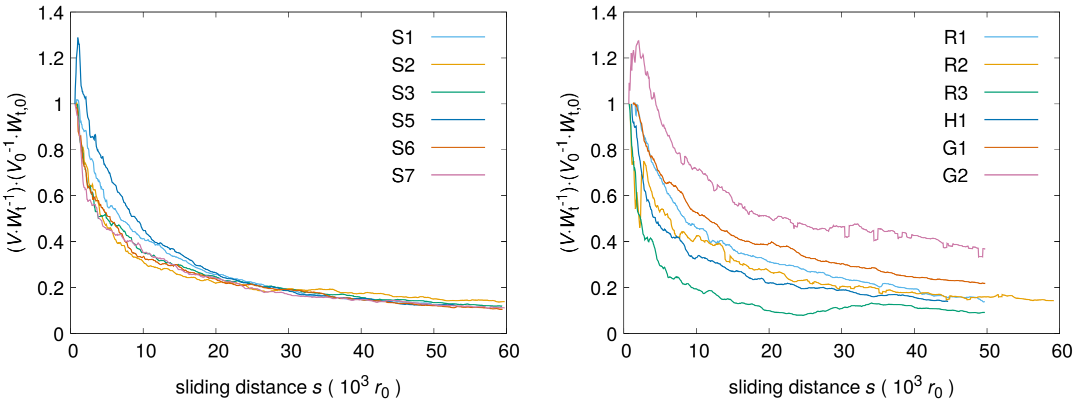

Supplementary Figure 7: Evolution of the ratio V/W_t between the wear volume V and the tangential work W_t with the sliding distance s. Left: single-asperity simulations, right: all other simulations. The ratio is normalized by the tangential work $W_{\mathrm{t},0}$ and volume V_0 produced during the formation of the wear particle. The ratio is not constant, hinting at a change in the behaviour of the wear volume evolution after running-in.

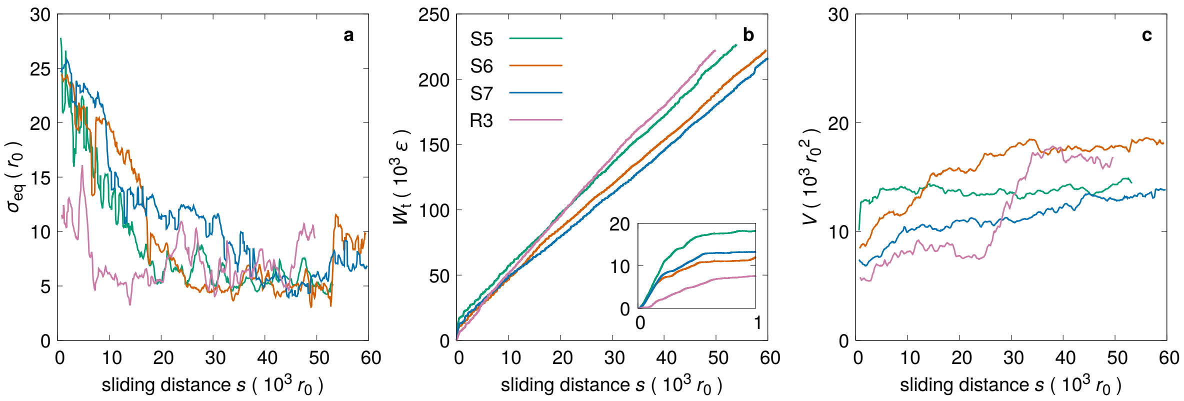

Supplementary Figure 8: Evolution of equivalent roughness σ_{eq}, frictional work W_{t}, and wear volume V. See Methods for the details on the definition of these quantities. See Figure 3 and Supplementary Fig. 9 for further simulations. a) Evolution of σ_{eq} for the composite surface for simulations S5, S6, S7, and R3 (see Table 1 for details). While for most simulations the value of σ_{eq} stabilizes, cold temperatures (S7) can slow down this stabilization (see Supplementary Discussion). b) Evolution of the tangential work W_{t} with the sliding distance. The work exhibits a sharp increase upon formation of the debris particle (inset)[1], after which the rate decreases and stabilizes. c) Evolution of the wear volume of the rolling debris particle, as defined only after its formation. In all simulated conditions the wear rate after the debris particle formation is small compared to the ratio of the initial particle size over the sliding distance necessary to form the particle (cf. Supplementary Fig. 6), consistent with the transition from severe to mild wear. In simulation R3, a significant temporary increase in the wear rate appears in the range of sliding distance $[25 \cdot 10^3; 35 \cdot 10^3]$ r_0. No significant change in σ_{eq}, W_{t} or F_{t} (cf. Supplementary Fig. 5) is linked to this change in volume, thus further investigation on its origin is needed.

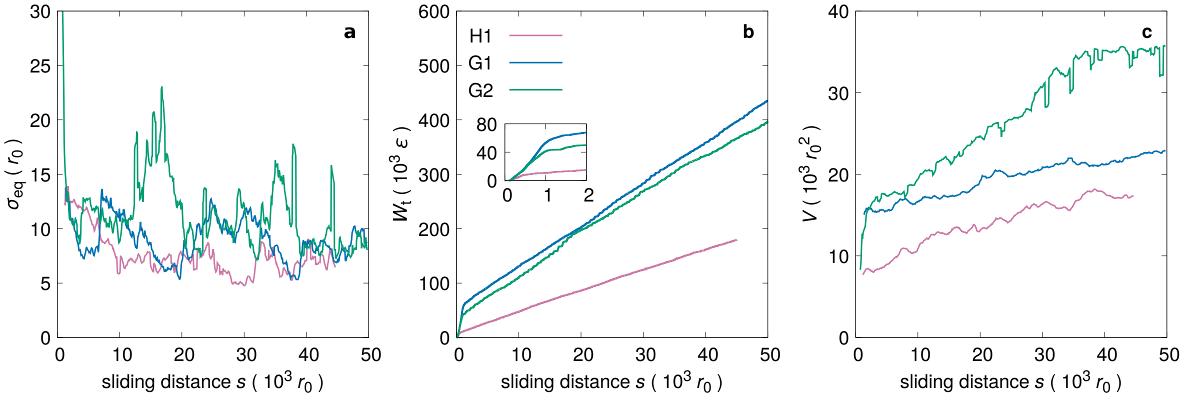

Supplementary Figure 9: Evolution of equivalent roughness σ_{eq}, frictional work W_{t}, and wear volume V. See Methods for the details on the definition of these quantities. Equivalent roughness σ_{eq}, frictional work W_{t}, and wear volume V for some representative simulations (see Methods for the details on how these quantities are defined). See Figure 3 and Supplementary Fig. 8 for further simulations. a) Evolution of σ_{eq} for the composite surface for simulations H1, G1, and G2 (see Table 1 for details). For simulation H1 the value of σ_{eq} stabilizes, and also for simulation G1 it appears to reach a steady-state, but with larger oscillations. A similar behaviour, with even larger oscillations, is observed for G2. The larger oscillations are a consequence of the presence of grain boundaries, which force the bulk to break along the boundaries or within the least tough potential, thus favouring the removal of chunks of material (cf. Supplementary Movie 1). This also leads to larger tangential work (b) input into the system. b) Evolution of the tangential work W_{t} with the sliding distance. The work exhibits a sharp increase upon formation of the debris particle (inset)[1], after which the rate decreases and stabilizes. c) Evolution of the wear volume of the rolling debris particle, as defined only after its formation. In all simulated conditions the wear rate after the debris particle formation is small compared to the ratio of the initial particle size over the sliding distance necessary to form the particle (cf. Supplementary Fig. 6), consistent with the transition from severe to mild wear[2].

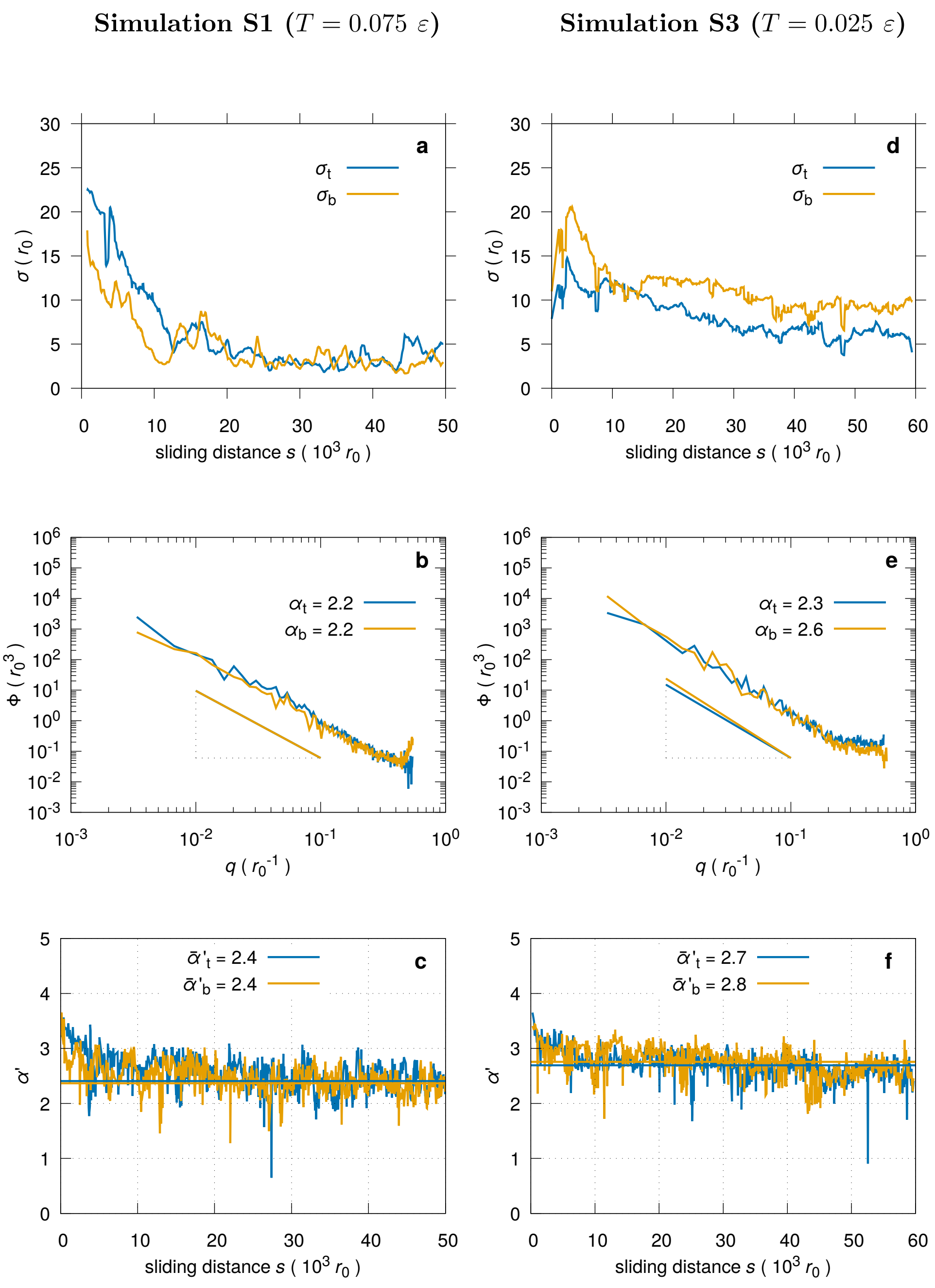

Supplementary Figure 10: Comparison between simulations run at different temperatures. Power spectrum analyses for top and bottom surfaces of simulations S1 (a–c) and S3 (d–f) are shown, where the main difference between the two is the temperature T (see Table 1). a) Evolution of σ shows that running-in takes place, but that is over within the first half of the simulation. b) PSD per unit length Φ of top and bottom surfaces averaged over ten different time steps in the stabilized state ($r_0 > 30\,000$). The straight segment represents a linear fit with exponents α_t and α_b. c) Linear fit exponent α' for the PSD during the whole simulation. The displayed means are averaged during the stabilized state only ($r_0 > 40\,000$) and have standard deviations 0.24 (top) and 0.20 (bottom). The values for the whole simulation are $\alpha'_t = 2.41 \pm 0.24$ and $\alpha'_t = 2.37 \pm 0.20$. d) No clear distinction between a transient phase and a more stable one appears for the colder case (see also Figure 3). e) PSD per unit length Φ of top and bottom surfaces averaged over ten different time steps ($50\,000 < r_0 < 60\,000$). The straight segments represent a linear fit with exponents α_t and α_b. f) Linear fit exponent α' for the PSD during the whole simulation. The displayed means are average values over the whole simulation with a standard deviation equal to 0.23 (top) and 0.26 (bottom).

Supplementary Discussion

Surface roughness evolution More insights are gathered by analysing the evolution of the surface roughness throughout the whole simulated sliding distances. Indeed, it has been observed experimentally that the adhesive wear process is characterized by two regimes, both in metals[2] and rocks[3]: a transient phase (or running-in) and a steady-state phase. The transient phase takes place at the beginning of the wear process and it is characterized by a decreasing wear rate, which at some point stabilizes, transitioning to the steady-state phase[2]. The change in the wear process is ascribed to the change in the morphology of the surfaces in contact[4,2,3]: the roughness of the surfaces (expressed in terms of centred line average roughness or root mean square of heights) is reduced or increased during the transient phase, until it reaches a stabilized value and a constant contact area, leading to a constant wear rate as described by Archard[5].

Figure 3 displays the evolution of the equivalent roughness σ_{eq}, of the work W_{t} performed by the tangential force, and of the volume V of the debris particle for some representative simulations (cf. Supplementary Fig. 8 and 9 for further simulations). Focussing first on the evolution of the roughness (see Figure 3a) it can be seen that the value of σ_{eq} undergoes a sharp increase upon debris particle formation, and then decreases, settling around the value $\sigma_{\mathrm{eq}} \approx 5\ r_0$ for most simulations. This is consistent with the abovementioned picture of a two-regime wear process. The value of σ_{eq} depends of the size of the system, and it is larger for larger systems (see Supplementary Fig. 2), as more wavelengths are involved in the description of the surface in the PSD.

The different behaviour observed in the simulation S3 is attributed to the lower temperature of the simulation, which is responsible for inhibiting plastic deformations and diffusion along the surfaces: this results in a more brittle behaviour and a longer running-in phase, which does not appear to finish within the investigated timescale. We thus take this simulation as a reference to investigate the transient phase of the adhesive wear process. We perform a linear fit of log(PSD) over the whole simulation and monitor the fitting parameter α' (Supplementary Fig. 10f). α' does not stabilize, consistent with the fact that no steady-state has been reached yet, and its average value is 2.7, which is larger than the value of 2.5 found during the steady-state (Supplementary Fig. 10c). Assuming that the surfaces are also fractal in the transient phase stage, the linear fitting parameter α' provides an estimate of the PSD scaling exponent, and the surfaces during running-in are thus expected to be rougher than at steady-state ($H \approx 0.85$).

The decreasing value for the linear fit coefficient α' and the steady-state value discussed above suggest a decrease of the Hurst exponent during the running-in, which in turn indicates an uneven decrease in the powers with the wavevectors, i.e. the different wavelengths forming the surface are smoothed in a different fashion. While further investigations are needed to accurately determine the origins of this behaviour, a possible explanation is that large wavelengths are smoothed proportionally more than short wavelengths. This is consistent with experimental findings in wear-polished cobblestone surfaces[6], where powers at long wavelengths undergo larger reduction than short ones, and may explain the large roughness reduction found at larger scales in experiments of rock against rock sliding contact[7]. Recalling the smoothing and re-roughening mechanism put forward in the main text, when a fragment is removed from a surface, it smooths the wavelengths larger than the fragment (and roughens those that are smaller). In the three-body configuration, the size of the contact between the debris particle and the opposing surface sets an upper limit for the dimension of newly formed fragments: wavelengths larger than the contact size are therefore expected to always smooth. Shorter wavelengths are smoothed and re-roughened continuously instead, hence the different smoothing rate (and the change in α'). We also observe that the large σ values in the early stage are due to the localization of the material removal in the surface upon the formation of debris particle; as the removed volume has a non-negligible size at the scale of our simulations, it is expected to affect the powers of the large wavelengths more. During the process that reduces σ, then, the power at large wavelengths decreases proportionally more than at short wavelengths, thus reducing the α' values.

Supplementary References

[1] Aghababaei, R., Warner, D. H. & Molinari, J.-F. On the debris-level origins of adhesive wear. *Proceedings of the National Academy of Sciences* **114**, 7935–7940 (2017).

[2] Queener, C., Smith, T. & Mitchell, W. Transient wear of machine parts. *Wear* **8**, 391–400 (1965).

[3] Wang, W. & Scholz, C. H. Wear processes during frictional sliding of rock: A theoretical and experimental study. *Journal of Geophysical Research: Solid Earth* **99**, 6789–6799 (1994).

[4] Kragelsky, I. V., Dobychin, M. N. & Kombalov, V. S. *Friction and wear: calculation methods* (Pergamon Press, 1981).

[5] Archard, J. Contact and rubbing of flat surfaces. *Journal of Applied Physics* **24**, 981–988 (1953).

[6] Persson, B., Albohr, O., Tartaglino, U., Volokitin, A. & Tosatti, E. On the nature of surface roughness with application to contact mechanics, sealing, rubber friction and adhesion. *Journal of Physics: Condensed Matter* **17**, R1 (2004).

[7] Davidesko, G., Sagy, A. & Hatzor, Y. H. Evolution of slip surface roughness through shear. *Geophysical Research Letters* **41**, 1492–1498 (2014).
