# Supplementary material for: Emergence of self-affine surfaces during adhesive wear
Source: Nat Commun. 2019 Mar 8;10:1116. doi: 10.1038/s41467-019-09127-8 (PMC6408517; doi:10.1038/s41467-019-09127-8)
Supplement: Supplementary file 3 — Description of Additional Supplementary Files [file 41467_2019_9127_MOESM3_ESM.docx]

**Description of Additional Supplementary Files**

File Name: Supplementary Movie 1

Description: The video is an example of the surface roughness evolution in our simulations. The video is of simulation G1, where heterogeneous material with grain boundaries is modelled. The top surface slides against the bottom one and at the beginning of the video (up to 2 s of playtime) contact takes place in a two body configuration. Asperities deform until a debris particle is formed (around 2 s). The system then switches to a three-body configuration, and the rolling particle works both surfaces. After a while, a steady-state for the roughness is reached. In this simulation (as in G2), the surfaces favour cracks within the least tough material (or at weak, heterogeneous interfaces), and plastic deformation within the toughest one. Colours distinguish particles originally belonging to the top (dark and light blue) and bottom (yellow and red) surfaces; black lines represent simulation box boundaries (periodic boundary conditions are applied along the horizontal direction).
